# Hydramnion

**Published:** 1887-05

**Authors:** 


HYDRAMNION (*excess of liquor amnii*).—Dr. W. B. Meaney, writing from London to *South Western Medical Gazette*, says that at Birmingham, May 21, 1886, Dr. Lawson Tait operated upon a case of hydramnion, mistaking it for an ovarian tumor, the patient having been brought to Dr. Tait's private hospital by a country physician, when upon examination it was decided to be an ovarian tumor.

Ether having been administered, Dr. Tait made his usual abdominal incision, when, on the introduction of his hand to break up the adhesion, it was found that the tumor, instead of being ovarian, was an enlarged uterus. An aspirator needle was introduced into the cavity of the uterus, and upon some of the fluid being withdrawn, it was discovered to be a case of hydramnion.

A Spencer Wells trocar was introduced, and about two and one-half gallons of the amniotic fluid drawn off. The uterus, with its contents, was brought out through the abdominal incision, when it was found to contain fœtal twins, showing the woman to have been in her fifth month of pregnancy. The neck of the uterus was transfixed with a clamp—the uterus cut off—the abdominal wound brought together by the aid of silk sutures in such a manner as to tightly embrace the neck of the uterus beneath the clamp. It was decided to allow the clamp to remain in its position until it should be removed by sloughing.

This was certainly a bold proceeding, and so far as we know, the first operation of the kind on record. It is not yet known what was the result of the case.

